# Editorial: Manifestations of mild-to-moderate traumatic brain injury

**DOI:** 10.3389/fnins.2023.1266355

**Published:** 2023-09-05

**Authors:** Steven H. Rauchman, Dimitris G. Placantonakis, Allison B. Reiss

**Affiliations:** ^1^Department of Neuroscience, The Fresno Institute of Neuroscience, Fresno, CA, United States; ^2^Department of Neurosurgery, NYU Grossman School of Medicine, New York, NY, United States; ^3^Department of Medicine, NYU Grossman Long Island School of Medicine, Mineola, NY, United States

**Keywords:** concussion, traumatic brain injury, white matter, treatment, diagnosis, experimental-animal models

## Introduction

Worldwide there are in excess of 27 million new traumatic brain injury (TBI) cases yearly, with over one million in the United States alone (The Lancet Neurology, 2022). The cumulative impact of these numbers on the individual, loved ones and society is magnified as symptoms often persist, emphasizing the importance of TBI as a Research Topic (Bowman et al., 2022). This Research Topic includes 8 diverse articles in several categories. There are 2 animal model papers that introduce proposed interventions. There are 5 papers that define function, anatomy, or cellular physiology of TBI. There is an additional paper covering aspiration, a serious complication of TBI.

## Murine studies assessing TBI

Schwab et al. examined cellular senesce after repeated experimentally induced mild TBI in a mouse model. 1 week after the last of 3 impacts, the injured mice displayed cognitive and behavioral abnormalities. Accumulation of DNA damage (manifested as double-strand breaks, oxidative lesions, and R-loops) was demonstrated near the region of impact as early as 1-week post trauma. Markers of cellular senescence were also noted at that timepoint. Intraperitoneal injection of the senolytic drug ABT263 reduced cellular senescence in male animals only. Results suggest future therapy should consider sex as a variable.

Bibineyshvili et al. explored sleep/wake cycle disturbances over 2 months in a blast injury model using C57BL/6 wildtype male mice. The rationale for the study was the role of sleep disruption in TBI sequelae in humans. In these mice, blast-induced injuries chronically disrupted memory and adversely affected performance of motor tasks. EEG/EMG activity showed TBI induced sleep disruption. Dexmedetomidine (DEX), a selective α-2 adrenoceptor agonist used in post-operative patients as a sedative and analgesic, was given randomly to one group of mice while the other group received saline. Mice were treated with DEX starting about 18 days after blast injury and DEX-treated mice showed statistically significant improvement in motor and cognitive function and reduced sleep disturbance with accompanying EEG improvements in intra-spindle frequency, theta and alpha power. Subcutaneous DEX administration requires investigation to establish value in human TBI.

## Function/anatomy/cellular physiology

Klimo et al. conducted a cross-sectional pilot study comparing retinal nerve fiber layer (RNFL) thickness between individuals with repeated TBIs and a control group. RNFL thickness reflects retinal ganglion cell function, which has been advanced as a non-invasive measure of a TBI. Spectral-domain optical coherence tomography (SD-OCT) and scanning laser polarimetry (SLP) were used to assess RNFL thickness. Electroretinography (ERG) was also a tested parameter. Importantly, there were no statistically significant differences in global RNFL thickness between TBI and control groups. There was not a statistically significant difference in photopic negative response amplitude, a central component of ERG testing between the 2 groups. In this pilot study, measurement of RNFL thickness by OCT did not distinguish TBI from control, but further studies are needed for a definitive conclusion.

Samadani et al. focused on whether the EyeBOX eye tracking algorithm provides a meaningful assessment of concussion. Previous research has demonstrated that TBI or concussion can disrupt neural pathways involving eye movement. Thus, eye tracking becomes a potential non-invasive screening device for concussion and TBI. This non-randomized study included 282 patients aged 5–67 from an emergency department with presumed concussion. The Sports Concussion Assessment Tool 3 (SCAT 3) was used to distinguish between presumptive true concussions and more mild head trauma. Higher scores were categorized into the concussion group and lower scores were considered non-concussed. Sensitivity of the EyeBOX test was 80.4 % and specificity was 66.1%. This was deemed acceptable for a heterogenous population. Eye tracking may be useful for screening head injury patients.

Abdullah et al. used diffusion MRI to study 11 adult males with non-severe TBI and 11 matched controls without TBI. 10 weeks post-injury, the difference in fractional anistrophy (FA) between groups was described and correlated to neuropsychological tests. There were multiple anatomic areas of the brain that demonstrated FA reductions compared to controls. Nine regions of the brain were defined as regions of interest (ROI). While some neuropsychological testing corresponded to known cognitive function in ROI, there were also some counterintuitive negative correlations. Overall, the study concludes that in non-severe TBI, there is FA data to support the hypothesis that white matters changes in the brain are related to brain injury. Evaluating white matter integrity may employ approaches beyond diffusion MRI, particularly, neurite orientation dispersion and density imaging (NODDI), which may be combined with diffusion MRI to yield more accurate metrics in mild TBI (Churchill et al., 2019).

Danielli et al. review traditional anatomic regions of the brain and each region's correlation to function. The distinction is made between white matter and gray matter areas of the brain. This anatomic/functional encyclopedia of brain regions attempts to explain the implications of head trauma on a global and specifically anatomic basis.

Rauchman et al. review the mechanisms and manifestations of TBI with emphasis on the visual system. Cellular response to TBI is examined which includes excitotoxicty with attention to the effects of high levels of glutamate released into the brain. Mitochondrial damage and oxidative stress are discussed. The broad and important topic of neuro-inflammation in TBI is recognized. Of clinical significance, the importance of the pupillary exam in evaluating TBI is emphasized. The subtle analysis of visual contrast sensitivity is summarized. A practical guide to mental and visual fatigue introduces an important element of patient-centered TBI assessment. A broad overview of potential interventions are characterized with the limits of current therapy.

## Preventing TBI complications

Han et al. conducted a randomized, single-blinded, controlled study of the utility of the Passy-Muir Tracheostomy and Ventilator Swallowing and Speaking Valve (PMV) in reducing aspiration as a complication in acquired brain injury (ABI). Before and after intervention swallowing biomechanics were evaluated using video fluoroscopy. Subglottic pressure increased in the group receiving PMV intervention compared with the non-PMV intervention group and this may be the mechanism reducing aspiration risk.

## Conclusions

The diagnosis and prognosis of TBI remain inexact (Ganti et al., 2019). The Glasgow Coma Scale (GCS) has traditionally been used to define the initial level of consciousness, but there is no simple screening test after head trauma (Fitzgerald et al., 2022). Routine brain scans are usually normal. GCS does not correlate tightly with outcomes. Current TBI research is multidimensional with no definitive intervention to improve outcome (Alves et al., 2019). Thus, the need for additional input is defined by this broad overview. Hopefully, this topic will motivate continued research.

## Author contributions

SR: Writing—original draft, Writing—review and editing. DP: Writing—review and editing. AR: Conceptualization, Writing—original draft, Writing—review and editing.
